# Diabetes and tuberculosis: the impact of the diabetes epidemic on tuberculosis incidence

**DOI:** 10.1186/1471-2458-7-234

**Published:** 2007-09-06

**Authors:** Catherine R Stevenson, Nita G Forouhi, Gojka Roglic, Brian G Williams, Jeremy A Lauer, Chirstopher Dye, Nigel Unwin

**Affiliations:** 1 Medical Research Council Epidemiology Unit, Cambridge, UK; 2 Diabetes Group, Department of Chronic Diseases and Health Promotion, World Health Organization, Geneva, Switzerland; 3 Stop TB, World Health Organization, Geneva, Switzerland; 4 Health Systems Financing, World Health Organization, Geneva, Switzerland; 5 Institute of Health and Society, Newcastle University, Newcastle upon Tyne, UK

## Abstract

**Background:**

Tuberculosis (TB) remains a major cause of mortality in developing countries, and in these countries diabetes prevalence is increasing rapidly. Diabetes increases the risk of TB. Our aim was to assess the potential impact of diabetes as a risk factor for incident pulmonary tuberculosis, using India as an example.

**Methods:**

We constructed an epidemiological model using data on tuberculosis incidence, diabetes prevalence, population structure, and relative risk of tuberculosis associated with diabetes. We evaluated the contribution made by diabetes to both tuberculosis incidence, and to the difference between tuberculosis incidence in urban and rural areas.

**Results:**

In India in 2000 there were an estimated 20.7 million adults with diabetes, and 900,000 incident adult cases of pulmonary tuberculosis. Our calculations suggest that diabetes accounts for 14.8% (uncertainty range 7.1% to 23.8%) of pulmonary tuberculosis and 20.2% (8.3% to 41.9%) of smear-positive (i.e. infectious) tuberculosis.

We estimate that the increased diabetes prevalence in urban areas is associated with a 15.2% greater smear-positive tuberculosis incidence in urban than rural areas – over a fifth of the estimated total difference.

**Conclusion:**

Diabetes makes a substantial contribution to the burden of incident tuberculosis in India, and the association is particularly strong for the infectious form of tuberculosis. The current diabetes epidemic may lead to a resurgence of tuberculosis in endemic regions, especially in urban areas. This potentially carries a risk of global spread with serious implications for tuberculosis control and the achievement of the United Nations Millennium Development Goals.

## Background

Tuberculosis remains a leading cause of death globally. In 2005 there were an estimated 8.8 million new cases of tuberculosis worldwide, with 1.9 million of those occurring in India[1]. Incidence of tuberculosis is greatest among those with conditions impairing immunity[2], such as human immunodeficiency virus (HIV) infection and diabetes. The consequences of mismanagement of tuberculosis in a patient with diabetes can be severe, but there are simple and immediate opportunities for improving treatment success and reducing mortality.

The global burden of diabetes is increasing, and recent estimates highlight the importance of this disease in India. There were an estimated 20–30 million people in India with diabetes in 2000 (estimates vary with study methodology) [3,4], and projections suggest prevalence will rise to almost 80 million people by 2030[4]. It is possible that in areas of high diabetes prevalence the impact of this diabetes epidemic[4] on tuberculosis could be as great as that of HIV[5], and the spread of HIV is one of the main reasons why targets set by the Stop TB Partnership (within the framework of the Millennium Development Goals) will not be met in several regions, at least at current rates of progress[6]. However, the overall importance of diabetes as a risk factor for tuberculosis is still largely unknown, although a recent analysis in Mexico concluded that, in the population studied, 25% of pulmonary tuberculosis was attributable to diabetes[5].

Our objective was to estimate the population-level impact of diabetes on the incidence of pulmonary tuberculosis in India. We chose India as an illustrative example because of its large population size, the availability of relatively good data on both diabetes and tuberculosis, and because the latter indicate that both these conditions are major public health problems there. We also aimed to evaluate the contribution made by diabetes to the higher tuberculosis incidence in urban compared with rural populations.

## Methods

### Epidemiological data

Data were extracted from the sources below, as summarised in Table 1. Analyses were limited to the adult population aged 25 years and over, as estimates of diabetes prevalence and the relative risks of incident tuberculosis associated with diabetes were available for this age group only.

**Table 1:** Summary of epidemiological data used to evaluate the importance of diabetes as a risk factor for tuberculosis in India in 2000 for adults aged 25 years and over

	**UN population estimate^12 ^(A)**	Diabetes prevalence*^3 ^% (B)	**Estimated number with diabetes (A × B)**	**Number of pulmonary tuberculosis^† ^incident cases^‡^**	**Number of smear-positive tuberculosis incident cases^‡^**	RR*^§ ^for pulmonary tuberculosis^†17 ^(95% CI)	RR*^§ ^for smear-positive tuberculosis^17 ^(95% CI)
Total	481,573,000	4.3	20,707,639	939,064	575,900	5.1 (1.7 – 15.8)	7.1 (2.9 – 17.2)
Women (age in years):							
25–29	40,462,000	2.1	849,702	59,738	34,834	7.8 (1.2 – 51.3)	6.6 (1.7 – 26.6)^||^
30–39	68,472,000	3.5	2,396,520	91,688	54,629	10.0 (6.8 – 14.5)	6.6 (1.7 – 26.6)^||^
40–49	50,913,000	4.7	2,392,911	49,159	31,084	4.7 (3.6 – 6.2)	12.7 (7.4 – 21.6)
50–59	35,327,000	5.1	1,801,677	28,220	17,710	2.3 (1.8 – 2.9)	5.2 (3.1 – 8.7)
60+	39,789,000	6.9	2,745,441	19,708	12,548	1.8 (1.1 – 2.9)	4.0 (1.4 – 11.4)
Men (age in years):							
25–29	43,998,000	2.1	923,958	108,527	63,283	7.8 (1.2 – 51.3)	6.6 (1.7 – 26.6)^||^
30–39	74,585,000	3.5	2,610,475	205,044	123,081	10.0 (6.8 – 14.5)	6.6 (1.7 – 26.6)^||^
40–49	55,434,000	4.7	2,605,398	166,705	105,841	4.7 (3.6 – 6.2)	12.7 (7.4 – 21.6)
50–59	35,796,000	5.1	1,825,596	118,990	74,500	2.3 (1.8 – 2.9)	5.2 (3.1 – 8.7)
60+	36,797,000	6.9	2,538,993	91,286	58,391	1.8 (1.1 – 2.9)	4.0 (1.4 – 11.4)

*Age-specific only; see Methods

^†^Smear-positive plus smear-negative

^‡^See Appendix for calculations

^§^Relative risk of incident TB associated with baseline diabetes

^||^As no RR was available for smear-positive TB incidence for the age band 25–29, the RR for 30–39 was used

#### Diabetes Prevalence

Several sources of diabetes prevalence data are available for India [3,7,11]; however, we used data from the Prevalence of Diabetes in India Study (PODIS)[3], as it was the largest study and the only one to ascertain urban and rural prevalences separately. PODIS is a population-based study of 18,363 participants (9,008 men, 9,355 women) aged ≥ 25 years in 77 centres throughout India. As PODIS reported no sex difference in crude diabetes prevalence, we applied the same age-specific prevalence estimates to men and women.

Numbers of diabetes cases in India were estimated by multiplying age-specific diabetes prevalence estimates from PODIS by the age- and sex-specific United Nations population estimates for India in 2000[12].

#### Tuberculosis Incidence

Tuberculosis case-notification data are routinely sent to the World Health Organization from the Revised National Tuberculosis Control Programme (RNTCP) in India. As the programme does not yet detect all new TB cases arising each year, the distribution of reported smear-positive cases by age and sex was used with the WHO crude estimate[1] (all ages, both sexes) of smear-positive tuberculosis incidence in India in 2000 to calculate the total number of smear-positive cases by age and sex. The percentage of new smear-positive cases occurring in each age/sex group was calculated by dividing the RNTCP-reported number of cases in each group by the total reported incidence.

RNTCP notifications only include information on age and sex for smear-positive cases. Age- and sex-specific incidences of total pulmonary tuberculosis (smear-positive plus smear-negative) were therefore calculated from the *estimates *for smear-positive incidences calculated previously. We adjusted for the difference in age distribution of smear-negative and smear-positive tuberculosis incidence by dividing the age- and sex-specific smear-positive incidence estimates by the age-specific ratios of smear-positive to total pulmonary tuberculosis[13]. We then calculated the overall incidence of total pulmonary tuberculosis in the population using the WHO estimate for total tuberculosis incidence in the population (smear-positive, smear-negative and extra-pulmonary)[1] and estimating that 20% of incident tuberculosis is extrapulmonary [14-16]. The adjusted age- and sex-specific incidences were then each multiplied by the ratio of the WHO-estimated crude incidence to the sum of the individual incidences, to ensure that the sum of the age- and sex-specific incidences was equal to the overall WHO crude incidence estimate.

#### Relative risk for tuberculosis associated with diabetes

We obtained age-specific relative risks for the association between diabetes and incident tuberculosis (for total pulmonary tuberculosis and smear-positive tuberculosis separately) from a study of 814,713 Korean civil servants[17]. As separate age-specific relative risks were not reported for men and women, we applied the same estimates to both sexes.

This is the only large-scale prospective study quantifying the diabetes-associated risk of incident tuberculosis (reactivation was excluded by baseline chest x-ray) within a single population. The only study we found at the time of undertaking the analyses which had been undertaken in India had a small sample size and was cross-sectional in design, so was less precise and could not account for temporal associations and does not provide age specific relative risk estimates,[18] Recently a case control study of risk factors for TB has been published from India in which known diabetes was ascertained by questionnaire[19], but this only provides a single, all ages, estimate of relative risk.

#### Population

Age- and sex-specific estimates of the resident Indian population in 2000 were obtained from UN World Population Prospects, 2004 revision[12].

### Statistical calculations

Estimates of diabetes prevalence, tuberculosis incidence and the relative risk of tuberculosis incidence associated with diabetes were applied to age- and sex-specific estimates of the Indian population to calculate the Attributable Fraction (Population)[20] (see below). We calculated crude estimates of the AF(P) using data for tuberculosis incidence and diabetes prevalence for the overall population, as well as estimates stratified by age and sex. The published 95% confidence intervals for the relative risks were used to derive upper and lower bounds for the AF(P) estimates.

We estimated the proportion of tuberculosis attributable to diabetes among those with diabetes using the Attributable Fraction (Exposed)[20] (see below) for smear-positive and total pulmonary tuberculosis. Age-adjusted relative risks and 95% confidence intervals were calculated using Mantel-Haenszel methods.

#### Attributable fractions: definitions and formulae

We used the following definitions and formulae for attributable fractions.

##### Attributable Fraction (Population)

The proportion by which the incidence rate of the outcome of interest (here, incident tuberculosis) in the *entire *population would theoretically be reduced if the exposure of interest (here, diabetes) were eliminated.

AF(P)=Pe(RR−1)1+Pe(RR−1)
 MathType@MTEF@5@5@+=feaafiart1ev1aaatCvAUfKttLearuWrP9MDH5MBPbIqV92AaeXatLxBI9gBaebbnrfifHhDYfgasaacH8akY=wiFfYdH8Gipec8Eeeu0xXdbba9frFj0=OqFfea0dXdd9vqai=hGuQ8kuc9pgc9s8qqaq=dirpe0xb9q8qiLsFr0=vr0=vr0dc8meaabaqaciaacaGaaeqabaqabeGadaaakeaacqWGbbqqcqWGgbGrcqGGOaakcqWGqbaucqGGPaqkcqGH9aqpdaWcaaqaaiabdcfaqnaaBaaaleaacqWGLbqzaeqaaOGaeiikaGIaemOuaiLaemOuaiLaeyOeI0IaeGymaeJaeiykaKcabaGaeGymaeJaey4kaSIaemiuaa1aaSbaaSqaaiabdwgaLbqabaGccqGGOaakcqWGsbGucqWGsbGucqGHsislcqaIXaqmcqGGPaqkaaaaaa@45C5@

where P_e _is the prevalence of the exposure and RR is the relative risk for the outcome of interest.

##### Attributable Fraction (Exposed)

The proportion by which the incidence rate of the outcome of interest (here, incident tuberculosis) in the *exposed *population would theoretically be reduced if the exposure of interest (here, diabetes) were eliminated.

AF(E)=RR−1RR
 MathType@MTEF@5@5@+=feaafiart1ev1aaatCvAUfKttLearuWrP9MDH5MBPbIqV92AaeXatLxBI9gBaebbnrfifHhDYfgasaacH8akY=wiFfYdH8Gipec8Eeeu0xXdbba9frFj0=OqFfea0dXdd9vqai=hGuQ8kuc9pgc9s8qqaq=dirpe0xb9q8qiLsFr0=vr0=vr0dc8meaabaqaciaacaGaaeqabaqabeGadaaakeaacqWGbbqqcqWGgbGrcqGGOaakcqWGfbqrcqGGPaqkcqGH9aqpdaWcaaqaaiabdkfasjabdkfasjabgkHiTiabigdaXaqaaiabdkfasjabdkfasbaaaaa@3938@

where RR is the relative risk for the outcome of interest.

#### Urban/rural distribution

We aimed to estimate the contribution made by diabetes to the higher tuberculosis incidence observed in urban as compared with rural populations. We approached this by calculating the theoretical urban and rural tuberculosis incidences which would be expected from the distribution of diabetes prevalence and the diabetes-associated relative risk for developing tuberculosis. This initially required partitioning tuberculosis incidence across the populations with and without diabetes. We defined the equation:

TB_T _= [TB_ND _× RR × P_DIA_] + [TB_ND _× (1 - P_DIA_)],

where TB_T _is the total tuberculosis incidence rate, TB_ND _is tuberculosis incidence in the sub-population without diabetes, RR is the relative risk of incident tuberculosis for diabetes, and P_DIA _is the prevalence of diabetes. This equation was used to estimate tuberculosis incidence in the populations with and without diabetes by solving mathematically for TB_ND_. Tuberculosis incidence in the population *with *diabetes is TB_ND _× RR. Theoretical urban and rural tuberculosis incidences were then calculated using the equation above with the urban and rural diabetes prevalences reported by PODIS[3]. For comparison, urban and rural tuberculosis incidences were also calculated using recent measurements of the annual risk of tuberculosis infection[21] and Styblo's equation[22], which has been shown to be applicable within India[23].

#### Prevalence of diabetes among tuberculosis patients

The prevalence of diabetes among people with tuberculosis was estimated by calculating the number of tuberculosis cases in the populations with and without diabetes, and hence the percentage of cases with diabetes. Numbers of tuberculosis cases were calculated by multiplying age-specific estimates of tuberculosis incidence and of diabetic and non-diabetic population size. Age-specific tuberculosis incidences in the populations with and without diabetes were estimated using the same methods described under *Urban/rural distribution*.

All calculations were carried out in Microsoft Excel 2003.

## Results

In India in 2000 there were an estimated 481,573,000 people over the age of 25 (12). Among these, 4.3% (20,707,639) had diabetes (3), and 939,064 developed pulmonary tuberculosis, of which 575,900 were smear-positive and hence infectious (Table 1).

### Population impact of diabetes on tuberculosis

We estimate that diabetes accounted for 14.8% (7.1% to 23.8% – upper and lower bounds based on relative risk 95% confidence intervals) of incident pulmonary tuberculosis in India in 2000, equating to 139,000 (67,000 to 224,000) cases. We estimated the proportion of incident smear-positive tuberculosis due to diabetes to be 20.2% (8.3% to 41.9%), or 116,000 (48,000 to 241,000) cases (Table 2).

**Table 2:** Fraction of tuberculosis attributable to diabetes in India in 2000 in the adult population aged 25 years and over

	Total pulmonary tuberculosis				Smear-positive tuberculosis			
		
	Attributable Fraction (Population) % (upper and lower bounds)		Excess cases	Percentage of excess cases	Attributable Fraction (Population) % (upper and lower bounds)		Excess cases	Percentage of excess cases
Overall (crude)	15.1	(2.8 – 38.9)	141,548	-	20.8	(7.7 – 41.0)	119,622	-
Overall (age-adjusted)	14.8	(7.1 – 23.8)	138,767	100.0	20.2	(8.3 – 41.9)	116,389	100.0
Women age in years:								
25–29	12.5	(0.4 – 51.4)	7,455	5.4	10.6	(1.3 – 35.0)	3,683	3.2
30–39	23.9	(17.0 – 32.2)	21,926	15.8	16.5	(2.2 – 47.3)	8,993	7.7
40–49	14.9	(10.9 – 19.7)	7,316	5.3	35.4	(23.2 – 49.2)	11,016	9.5
50–59	6.2	(4.1 – 8.8)	1,755	1.3	17.6	(9.5 – 28.3)	3,112	2.7
60+	5.0	(0.5 – 11.6)	982	0.7	17.0	(2.6 – 41.7)	2,128	1.8
Men age in years:								
25–29	12.5	(0.4 – 51.4)	13,544	9.8	10.6	(1.3 – 35.0)	6,691	5.7
30–39	23.9	(17.0 – 32.2)	49,034	35.3	16.5	(2.2 – 47.3)	20,261	17.4
40–49	14.9	(10.9 – 19.7)	24,809	17.9	35.4	(23.2 – 49.2)	37,511	32.2
50–59	6.2	(4.1 – 8.8)	7,398	5.3	17.6	(9.5 – 28.3)	13,091	11.2
60+	5.0	(0.5 – 11.6)	4,548	3.3	17.0	(2.6 – 41.7)	9,903	8.5

In the sub-population of the estimated 20.7 million adults with diabetes in India, our calculations indicate that diabetes accounts for 80.5% (39.9% to 93.7%) of the 172,000 annual incident cases of pulmonary tuberculosis, and 85.9% (65.9% to 94.2%) of the 135,000 cases of smear-positive (i.e. infectious) tuberculosis (Table 3).

**Table 3:** Proportion of tuberculosis attributable to diabetes in the subpopulation of people with diabetes

	Attributable Fraction (Exposed)			
	
	Total pulmonary tuberculosis %(upper and lower bounds)		Smear-positive tuberculosis % (upper and lower bounds)	
Overall	80.5	(39.9 – 93.7)	85.9	(65.9 – 94.2)
Age in years				
25–29	87.2	(15.3 – 98.1)	84.9*	(39.4 – 96.2)
30–39	90.0	(85.4 – 93.1)	84.9*	(39.4 – 96.2)
40–49	78.8	(72.1 – 83.9)	92.1	(86.6 – 95.4)
50–59	56.5	(45.4 – 65.5)	80.7	(67.4 – 88.5)
60+	43.2	(6.5 – 65.6)	74.7	(27.5 – 91.2)

*As no relative risk (RR) was available for smear-positive tuberculosis incidence for the age band 25–29, the RR for the age band 30–39 was used.

### Urban/Rural differences

Our calculations suggest that the increased prevalence of diabetes in urban areas is associated with a 15.2% greater smear-positive tuberculosis incidence and a 10.8% greater total pulmonary tuberculosis incidence in urban compared with rural areas. We estimate that the incidence of smear-positive tuberculosis in urban areas is in fact 69.2% greater than that in rural areas, from calculations using measurements of the annual risk of tuberculosis infection, suggesting that diabetes accounts for approximately a fifth of the total difference.

### Prevalence of diabetes among tuberculosis patients

We predict that in India 18.4% (12.5% to 29.9%) of people with pulmonary tuberculosis (both smear-positive and smear-negative) have diabetes, and that in the smear-positive group diabetes prevalence is 23.5% (12.1% to 44%).

## Discussion

Our findings suggest that a substantial proportion of incident tuberculosis in India is attributable to diabetes;14.8% of pulmonary tuberculosis and 20.2% of smear-positive – i.e. infectious – tuberculosis. They also suggest that diabetes is present in 18.4% of adults in India with pulmonary tuberculosis and in 23.5% of those with smear-positive tuberculosis, despite a national adult diabetes prevalence of 4.3%. This result is comparable to that of a recent study in Mexico, which found a diabetes prevalence of 35% among tuberculosis patients in a district with an adult diabetes prevalence of 5.3%[5].

Estimates of the urban/rural distribution of the annual risk of tuberculosis infection suggest that, on average, smear-positive tuberculosis incidence in India is 69.2% higher in urban compared with rural areas. Crowded living conditions in urban districts are one possible factor. However, the increased prevalence of diabetes in urban areas may also play a role – according to our calculations, diabetes is responsible for the urban incidence of smear-positive tuberculosis being 15.2% greater than that in rural areas, or approximately a fifth of the total difference. Our results suggest therefore that the increased diabetes prevalence associated with the rapid urbanization taking place in India has important implications for tuberculosis control.

Our findings are subject to the general caveats applied to attributable risk estimates, for example that we assume a causal association, that other risk factors for tuberculosis are equally distributed across those with and without diabetes, and that those made more susceptible to infection by diabetes are fully exposed to the tuberculosis risk. One underlying risk factor for tuberculosis that may not be equally distributed between those with and without diabetes in India is poverty. Consistent with this a recent case control study from India of risk factors for TB found a univariate odds ratio of 1.8 for previously diagnosed diabetes, which strengthened to 2.44 when controlling for other risk factors, including low socio economic status[19]. However, even allowing for an uneven distribution in other risk factors between those with and without diabetes our attributable risk estimates may well be conservative because our prevalence figures for diabetes are conservative. A large study measuring the prevalence of diabetes in urban areas in India reported that 12.1% of adults had diabetes[10], compared with an urban prevalence of 5.6% found by the study used in our calculations[3]. Recalculating the Attributable Fraction (Population) using this higher value suggests that in urban areas this could be as high as 33.3% (7.4% to 64.2%) for pulmonary tuberculosis and 42.5% (19.0% to 66.2%) for smear-positive tuberculosis. Additionally, we have not considered the contribution to tuberculosis risk from hyperglycaemia below the diabetic threshold. Published data on the association between non-diabetic hyperglycaemia and tuberculosis are rare. However, a recent case control study from Indonesia[24] reported an odds ratio for the risk of tuberculosis associated with impaired fasting glucose (4.2, 95% CIs 1.5–11.7) as similar to that for diabetes (4.7, 2.7 – 8.1). The prevalence of impaired fasting glucose and of impaired glucose tolerance tend to be similar to or higher than the prevalence of diabetes[8,10], and thus the overall impact of hyperglycaemia may be even higher than our estimates presented here suggest. Population-level measures for managing hyperglycaemia may potentially be cost-effective simply in terms of their benefit to tuberculosis control.

### Limitations and strengths

A consequence of using separate studies for the different estimates used in our calculations is an inability to account for the inherent biases of each contributing study. However, so long as each of the studies is independently valid, this does not invalidate our conclusions as long as the assumptions involved are clearly understood.

In deciding on which study to use for the relative risk estimates we searched thoroughly for studies describing the association between tuberculosis and diabetes, and have critically reviewed these studies elsewhere [25]. There is consistent evidence from a number of studies, with different designs and from geographically diverse areas that diabetes is associated with an increased risk of tuberculosis, with an overall increased risk around 1.5 to 8 times higher. However, there are several limitations in the published studies, concerning in particular sample size, the case definitions used for diabetes and tuberculosis, the assessment and control for potential confounders and the fact that most do not provide age specific relative risks or odds ratios[25].

We chose to use relative risk estimates from the study in Korea[17] for several reasons. Firstly, the lack of robust studies reporting age specific relative risk estimates on the association between diabetes and TB from India meant that we had to look elsewhere. Secondly, the study from Korea is the only genuine prospective cohort study on this topic in the past 20 years, and thirdly it is one of only two studies we found that provided age specific relative risk estimates. In addition, based on chest X-rays at baseline the study was able exclude reactivation of pulmonary TB and assess the association of diabetes with new cases. It is, however, important to acknowledge the study's shortcomings. In particular the definition of diabetes was based on unconventional glucose cut points (i.e. 150 mg/dl for fasting and 180 mg/dl post prandial – as opposed to 140 and 200 mg/dl respectively as recommended by WHO at that time). In addition, the diagnosis of diabetes was based on glucose measurement at one point in time, rather than repeated measurements to confirm the diagnosis. This is common to virtually all epidemiological studies of diabetes but is likely to result in significant misclassification of cases of diabetes due to a mixture of biological variation in blood glucose levels and measurement error. It is likely that this led to an underestimate of the association between diabetes and tuberculosis. The crude prevalence of diabetes was low, being 1.2% in men and only 0.2% in women, and there were only 3 women with diabetes (out of 320) who developed TB, and thus sex and age specific relative risk estimates were not available. A further limitation is that there are likely to be underlying confounding factors that we have not accounted for. One of these is smoking, which is implicated as a risk factor for both diabetes and tuberculosis. Further work could include adjusting the diabetes-associated risk of tuberculosis incidence for the effect of smoking.

Nonetheless the study was large and well-structured, similar age specific relative risk estimates were found by a group working in Mexico[5], and the physiological mechanisms underlying diabetes-associated susceptibility are unlikely to vary between populations.

Our finding that diabetes is more strongly related to smear positive than smear negative TB reflects the greater relative risks of diabetes for this form of TB found in the study from Korea (see table 1). This relatively greater association between diabetes and smear positive TB compared to smear negative pulmonary TB, has been found in most, but not all, studies that have addressed this issue [25]

The strengths of our study are that the estimates used are taken from reliable, published sources, chosen after a consideration of the available options, and we explore a new hypothesis using a straightforward and transparent method. Further, our study represents the first attempt we are aware of to quantify the population impact of diabetes on tuberculosis in India.

### Implications

Currently, the future impact of tuberculosis control programmes is predicted from knowledge of the effects of chemotherapy and how it is modified by the HIV epidemic. The findings we report indicate that diabetes also has a considerable effect on tuberculosis epidemiology, and so it is important to adapt tuberculosis programme forecasts to incorporate additional risk factors.

The importance of the association between diabetes and tuberculosis is highlighted by the immediate relevance to the UN Millennium Development Goals, as it offers opportunities for reducing the death rate from tuberculosis, and improving its detection and treatment. It is widely recognised that HIV makes a substantial contribution to the global tuberculosis crisis. It is also known that cooperation to target HIV and tuberculosis simultaneously is crucial for the control of both diseases. In India, HIV accounts for 3.4% of adult tuberculosis incidence[2]; the proportion we estimate to be attributable to diabetes is 14.8%. The impact of diabetes on tuberculosis is therefore already considerable, and the predictions of a diabetes epidemic suggest this is likely to escalate.

In the past, an association between tuberculosis and diabetes was widely accepted. Indeed, half a century ago expert clinics were established for "tuberculous diabetics" and appeared to be successful in reducing the otherwise high mortality rate[26]. Today, however, the potential public health and clinical importance of this relationship seems to be largely ignored. For example, national clinical and policy guidance in the UK on the control of tuberculosis does not consider the relationship with diabetes[27]. The World Health Organization's new "Stop TB Strategy" refers to the problem of TB in "high-risk groups" including people with diabetes[28], but WHO has not yet made specific recommendations concerning the relationship between the two conditions. The recently published international standards for TB care give only cursory mention to diabetes [29,30]. There are, however, some guidelines, such as those from American Thoracic Society[31], which explicitly recommend screening for latent tuberculosis in patients with diabetes and a low threshold of investigation for tuberculosis in people with diabetes with unexplained symptoms. There is a need for new research to guide policy and practice in this area. This includes the need for robust studies of the association between the two conditions, particularly from parts of the world such as India where diabetes is increasing rapidly and TB remains highly endemic. There is evidence that people with TB and diabetes have worse TB outcomes than those without diabetes[25]. For example, a study from Indonesia found that people with diabetes are more than twice as likely to remain sputum culture positive at the end of treatment[32]. The potential impact of diabetes on the success of TB treatment and hence appropriate treatment strategies for those with the two diseases deserves investigation in other parts of the world. Another area worthy of investigation is the potential cost effectiveness of screening people with diabetes for TB in highly endemic areas where diabetes is now common.

## Conclusion

We have illustrated, using data from India, that diabetes makes a substantial contribution to tuberculosis incidence. The current diabetes epidemic may lead to a resurgence of tuberculosis in endemic regions, especially in urban areas. This has potentially serious implications for tuberculosis control, and it must become a priority to use this knowledge to initiate focused and coordinated action, including new research in parts of the world where diabetes is epidemic and TB endemic to properly inform public health and clinical practice. It is time that the "unhealthy partnership" [33]of tuberculosis and diabetes receives the attention it deserves.

## Competing interests

The author(s) declare that they have no competing interests.

## Authors' contributions

NU developed the original idea for this work, worked closely with CS on detailed aspects of the paper, and contributed to its writing and editing. CS extended the initial analysis design, carried out the calculations, wrote the first draft, and collated the responses from the other authors. NF worked closely with CS to develop the analytical methods and contributed to writing and revising the article. JL and GR contributed to the design of the study, to developing the methods and to revising the article. BW contributed to the initial study design and to revising the article. CD contributed to revising the article. All authors approved the final version for publication.

## Pre-publication history

The pre-publication history for this paper can be accessed here:


